# PI3K Functions in Cancer Progression, Anticancer Immunity and Immune Evasion by Tumors

**DOI:** 10.1155/2011/947858

**Published:** 2011-10-20

**Authors:** Francesco Dituri, Antonio Mazzocca, Gianluigi Giannelli, Salvatore Antonaci

**Affiliations:** Department of Emergency and Organ Transplantation, Section of Internal Medicine, Allergology and Clinical Immunology, University of Bari Medical School, 70124 Bari, Italy

## Abstract

The immunological surveillance of tumors relies on a specific recognition of cancer cells and their associate antigens by leucocytes of innate and adaptive immune responses. However, a dysregulated cytokine release can lead to, or be associated with, a failure in cell-cell recognition, thus, allowing cancer cells to evade the killing system. The phosphatidylinositol 3-kinase (PI3K) pathway regulates multiple cellular processes which underlie immune responses against pathogens or malignant cells. Conversely, there is accumulating evidence that the PI3K pathway is involved in the development of several malignant traits of cancer cells as well as their escape from immunity. Herein, we review the counteracting roles of PI3K not only in antitumor immune response but also in the mechanisms that cancer cells use to avoid leukocyte attack. In addition, we discuss, from antitumor immunological point of view, the potential benefits and disadvantages arising from use of anticancer pharmacological agents targeting the PI3K pathway.

## 1. PI3K Pathway in Tumor Development and Progression

**Figure 1:**
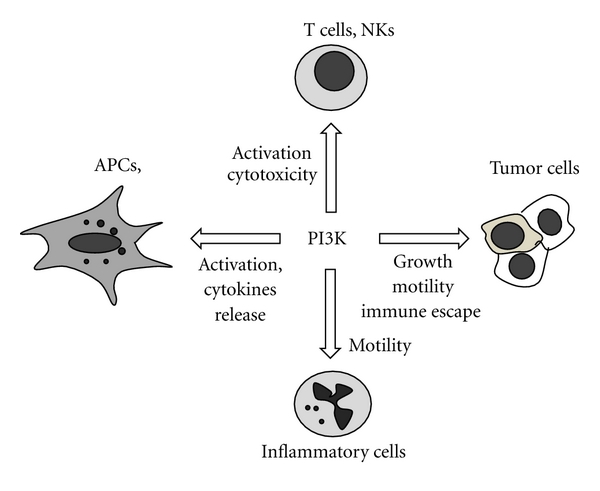
Schematic model of the PI3K signaling pathway involved in the regulation of a broad range of cellular activities in both immune system and cancer.

The PI3K signaling pathway regulates the activities of a broad range of downstream molecular effectors, which in turn act synergistically to mediate a number of cell behaviors and properties in both normal and pathological conditions. An overview of the involvement of PI3K in these conditions is summarized in Figure 1. Three classes of PI3K enzymes have been defined. The class I is the most intensely studied and includes p110*α*, *β*, *γ*, and *δ* catalytic isoforms, which are controlled by coupling with their proper regulatory isoforms (p85 and p101) to effect their lipid kinase activity [1]. The PI3K activation in terms of signaling response varies according to the type of stimulus. For example, p110*α* and *δ* are recruited and activated at the plasma membrane upon activation of tyrosine kinase receptors (TKRs) whereas p110*γ* requires engagement of G-protein-coupled receptors (GPCR). Conversely, p110*β* can be activated by both TKRs and GPCR [2]. Nevertheless, recent data reveal a more complex regulation for p110*δ*, as this isoform is linked to specific GPCRs signaling [3]. Once activated, PI3K enzymes catalyze the phosphorylation in position 3 of the inositol ring of phosphoinositides, resulting in the generation of 3-phosphoinositides, mainly the phosphatidylinositol-3-trisphosphate (PIP3). These lipids act as docking sites for the recruitment at plasma membrane of protein-bearing pleckstrin homology (PH) domain such as Akt/PKB, PDK1, BTK, and PLC*γ*. Once bound to PIP3 lipids, these proteins turn activated and signal to a wide array of downstream effectors that ultimately leads to multiple cellular responses [4–8]. This signaling cascade can be antagonized by the action of the phosphatase and tensin homolog (PTEN), a widely recognized tumor suppressor which dephosphorylates the PIP3 [9].

The oncogenic transformation of cultured cells as well as the progression of a variety of tumors *in vivo* has been reported to be induced by mutations or overexpression of p110 isoforms. For example, cultured cells undergo transformation when a catalytically hyperactive mutated isoform of p110*α* is ectopically expressed whereas p110*β*, *γ*, and *δ* are oncogenic only when overexpressed [10]. Mutations of p110*α* disrupting the interaction with the p85 subunit can also induce oncogenic transformation in the absence of the receptor activation [11, 12]. The oncogenic role of p110*α* has been previously demonstrated in ovarian cancers, where an increased number of PIK3CA gene copies was observed. This was correlated with the overexpression of the p110*α* subunit that results in an augmented activity of PI3 kinase [13]. Mutations of the PIK3CA gene were found with high frequency in colon, brain, breast, liver, and gastric cancers suggesting an involvement of isoform p110*α* in cancer [14, 15]. The activity of p110*β*, but not p110*α*, was shown to be essential in promoting PTEN-driven tumorigenesis in an animal model of prostate tumor. Importantly, Akt is shown to be a mediator of p110*β*-dependent tumorigenesis [16]. This finding was supported by a complementary approach based on the transgenic expression of a constitutively activated p110*β* in prostate of mice. In this study, overexpression of this hyperactive isoform drives the formation of a intraepithelial neoplasia [17]. p110*γ* has been recently shown to positively regulate tumor cell proliferation in HCC and pancreas cancer [18, 19]. In addition, pharmacological inhibition of p110*γ* in medulloblastoma cell lines led to an impairment in cell proliferation and sensitized them to cisplatin treatment [20]. A role for p110*δ* in sustaining neuroblastoma growth has been recently reported. Both primary neuroblastoma cells and tissues displayed an overexpression of p110*δ* and p85*α* in comparison with the normal adrenal gland tissue. Moreover, knockdown of both p110*α* and *δ* isoform triggered defective cell growth, whereas only p110*δ* knockdown affected cell survival, via lowering the expression of the Bcl-2 antiapoptotic family proteins [21]. The progression of multiple B-cell malignancies was found to be dependent on a constitutive activation of p110*δ* [22]. In particular, increased levels of p110*δ* were found in blast cells from patients with acute myeloid leukemia (AML). In addition, pharmacological targeting of p110*δ* resulted in an inhibition of the AML cell proliferation [23]. Finally, the PI3K signaling pathway was shown to be constitutively activated in chronic lymphocytic leukemia B cells (CLL). Moreover, dysregulation of the PI3K signaling pathway prevents CLL cell survival by inducing apoptosis through caspase-3 activation [24].

## 2. Role of PI3K Pathway in Immune Response to Tumors

Different cell types are involved in immune response to tumors. Natural killer (NK) cells intervene in a first-line defense against tumor cells. These lymphocytes constantly comb the cell microenvironment, where they check the expression level of MHC class I at the membrane of their targets, which can be reduced as a result of viral infection or oncogenic transformation. NK cells are cytotoxic against cells that fail to expose MHC class I on their surface, thanks to NK-inhibiting receptors for MHC class I that exist on cell membrane of NK cells [25]. Once activated, these receptors (belonging to three families named KIRs, ILTs, and NKG2A/CD94) inhibit for the cytolytic activity of NK cells by binding to HLA class I. Beside inhibitory receptors, NK cells bear different activating receptors which elicit their cytolytic effect on target cells after binding to a broad range of ligands. One of the best studied among the activating receptors of NK cells is the C-type lectin-like superfamily member NKG2D, which also occurs in CD8 T cell in humans. This receptor is a transmembrane glycoprotein which binds some known ligands (MHC class I chain-related molecules (MIC) MIC-A, -B, and ULBP) which are little expressed on the surface of normal cells but can be increased in transformed or virus-infected cells [26]. The antigen-presenting cells (APCs), primarily dendritic cells (DCs) and macrophages, can prime specific CD4+ and CD8+ T-lymphocyte-mediated responses to cancer cells, thanks to their ability to recognize tumor-associated or specific antigens, and present antigen-derived peptides in the MHC class II. The generation of tumor addressed T-cell clones is driven by stimulatory signals occurring when immunological synapses form between APCs and T-cells. DCs and macrophages secrete cytokines, such as IL-12, IL-15, IL-18, necessary for induction of NK and T-cell immunity. IL-12 leads to differentiation of CD4+ cells in Th1 subtype which is effective in tumor rejection. Th1 cells help expand the population of CD8+ cytotoxic T lymphocytes that can directly destroy tumor cells [27]. NK cells release IFN*γ* in response to stimulation by both mature DCs secreted IL-12 and cell-to-cell contact with DCs [28]. Also, IL-12 stimulate Th1 and CD8+ to secrete IFN*γ* [29] which in turn promotes a wide array of host responses to tumors [30], including the activation of CD8+ cells [31] and the recruitment of NK cells within the tumor [32].

Chronic inflammation is thought to underlie the onset of several cancers. Several reports demonstrate that PI3Ks activity is essential in regulating chemokine production by leukocytes as well as directional migration of these cells during the inflammatory response. For example, studies carried out *in vivo* using models for inflammation show that p110*γ* is required to allow chemotactic migration of neutrophils, macrophages, and effector CD8 T cells to inflammatory sites [33, 34]. During lung inflammation, recruitment of eosinophils to the bronchial epithelium, together with the repulsion of neutrophils exerted by chemokine gradients rely on the activity status of PI3K signaling in these leukocytes [35]. Moreover, the release of IL-8, Mip-1*α*, and Mip-1*β* by neutrophils in response to LPS and TNF*α* require the activity of p85/p110*δ* complex [36].

Studies performed in mice using loss of function of p110 isoforms and their related regulatory subunits demonstrate a crucial role for PI3K in development of immune cells involved in tumor clearance. The PI3K/Akt-dependent mTOR pathway is reported to be essential in GM-CSF-induced differentiation of DCs from monocytes [37]. Webb et al. demonstrate that the functions of p110*γ* and p110*δ* PI3K isoforms are required for T-cell development [38]. In a study recently published, Kerr and Colucci report the need for p110*δ* to achieve NK cell maturity, as well as a cooperation between p110*γ* and p110*δ* isoforms in establishing the repertoire of inhibitory receptors of the Ly49 family in mice (the homolog family in humans is KIR) [39]. Other authors have previously shown that the achievement of NK cell subsets maturity is impaired in mice either expressing lipid kinase-inactive p110*δ* or lacking regulatory p85*α*/p55*α*/p50*α* subunits. Moreover, inactive p110*δ* or p85*α*/p55*α*/p50*α* depletion was shown to result in significantly compromised NKG2D, Ly49D, and NK1.1 receptor-mediated cytokine and chemokine generation in NK cells, even if the NK-mediated cytotoxicity against tumor cells was affected only in mice lacking p85 regulatory subunit [40, 41].

An involvement of the PI3K/Akt pathway has been reported in the immune recognition of tumor cells. For example, in NK cells, the NKG2D-associated adapter protein DAP10 undergoes Tyr phosphorylation in its cytoplasmic tail following interaction between NKG2D and activating ligands. This allows DAP10 to anchor to either the p85 subunit of PI3K or to the adaptor Grb2, leading to PKB/AKT or MAP kinase signaling activation, respectively. These signaling cascades enable cytolytic activity and chemokine production by NK cells [42–44]. Furthermore, the small Ras family GTPase Rap1 is activated downstream of NKG2D engagement in a PI3K- and CrkL-dependent manner and is required for NK cell/target cell conjugate formation, NK cell polarization, and NKG2D-dependent cellular cytotoxicity [45]. Different activating receptors, other than NKG2D, can lead to NK cytotoxicity against tumor cells using the adapter DAP12, instead of DAP10, for PI3K pathway stimulation. DAP12 is tyrosine phosphorylated upon tumor cell ligation allowing binding of DAP12 to Syk kinase, which in turn activates the signaling pathway PI3K, Rac1, PAK1, and ERK leading to the lytic cascade of NK cells [46].

The engagement of NKG2D through coculturing human NK cells with MICA-bearing tumor cells leads to a PI3K-dependent increase of IFN*γ* secretion by NK cells. This is an additional effect to IFN*γ* release upon treatment of the same cells with IFN-*α*, IL-12, and specific agonists for TLR3- and TLR7-activating receptors [47]. These findings support the relevant role of the PI3K pathway as a mediator of the adaptive immune response against tumors by activated NK cells. The role of PI3K in the APCs production of IL-12 remains controversial. A report by Ohtani and coworkers show a complex cooperation between the PI3K-downstream GSK3 and mTOR pathways in the regulation of IL-12 secretion as a consequence of TLR activation by LPS on DCs. These authors show that GSK-3 and mTOR activities promote and reduce IL-12 production, respectively. However, the overall effect of LPS on DCs is to reduce IL-12 secretion, since PI3K activation blocks GSK-3 function while enhancing the mTOR signaling [48]. Conversely, other studies show an overall increased IL-12 production by human macrophages and DCs, upon LPS stimulation which depends on the activation of p110*β* isoform of PI3K [49].

The CD28-dependent costimulating signals required for the full activation of T cells by APCs are mediated partially by PI3K functions. CD28 undergoes tyrosine phosphorylation in its cytoplasmic tail upon binding to APCs costimulatory ligand B7. This binding recruits p85 subunit at the cell membrane through the interaction between SH2 domains of p85 and the phospho-tyr docking sites of CD28. As a consequence, p85 binds to the catalytic subunit p110 that activates PKC*θ*, which is capable of preventing stress-induced apoptosis of T cells [50].

## 3. The PI3K/Akt Pathway Is Involved in Escape of Tumors from Immunological Surveillance, Immune Suppression, and Acquired Leukocyte-Like Properties by Cancer Cells

**Figure 2:**
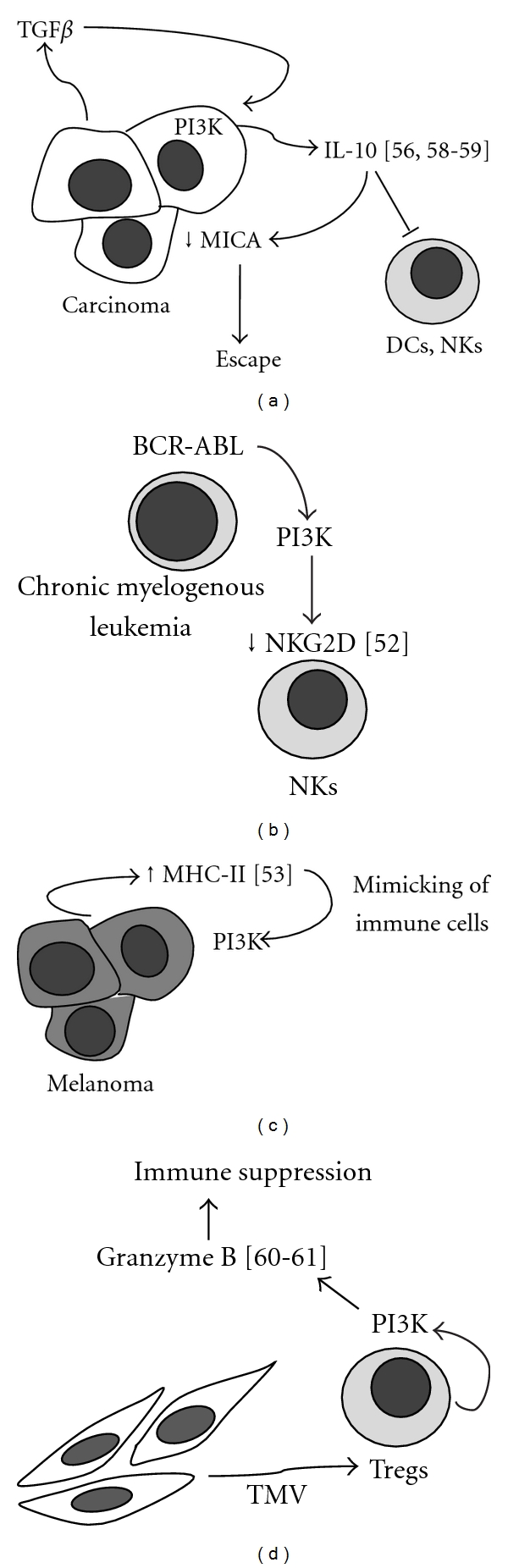
Examples of the major immune escape mechanisms of different types of cancers displaying the involvement of the PI3K signaling pathway. ↑: upregulation; ↓: downregulation; →: activation/secretion; *⊤*: inhibition.

The PI3K pathway can be responsible, to a certain extent, for transformed cells escaping immunity. Examples of some of the immune escape mechanisms by cancer involving the PI3K signaling pathway is summarized in Figure 2. A reduced NKG2D expression and function in NK cells following chronic exposure to NKG2D ligands and/or soluble forms of MIC (sMIC) leads to a immune surveillance failure [51]. This occurs in chronic myeloid leukemia, where the BCR/ABL fusion oncoprotein is shown to positively regulate the expression of MICA/B at the translational level via a PI3K-dependent mechanism in the BCR/ABL+ cell line K562 [52]. Cancer cells can also escape immune surveillance by developing a *de novo* expression on their surface of some molecules which are normally present in immune cells, thus allowing them to be recognized as normal. Melanoma cells often express MHC II, and this histological condition is associated with poor prognosis. Melanoma-infiltrating T cells express the lymphocyte activation gene 3 (*LAG-3*), which is a natural ligand for MHC II. Activation of MHC II on melanoma cells promotes resistance against FAS-mediated or drug-induced apoptosis via a mechanism based on MAPK/Erk and PI3K/Akt pathways [53]. Noh and coworkers supported furthermore the role of PI3K/Akt axis in the setting of immune escape. An immune-resistant human papillomavirus type 16 (HPV-16) E7-expressing tumor cell line was generated by these authors. A hyperactivation of Akt, after E7-specific vaccine administration, was found to be responsible for the increased resistance of these cells to CD8(+) T-cell-mediated apoptosis [54].

In addition, cancer can overcome immunity through a metabolic enhancement arising from *de novo* expression of pathways that leukocytes use in anticancer processes. Unexpectedly, a *de novo* expression of the NKG2D/DAP10 complex has been reported in human cancer cells both *in vitro* and *in vivo*. Notably, in this study, the authors demonstrate a complementary function between NKG2D/DAP10 and its MICA ligand, resulting in a self-sufficiency of cancer cells in activating of PI3K/Akt-dependent NKG2D downstream signaling. Therefore, the activation of Akt-downstream mTOR/S6K/4EBP1 signaling axis upon NKG2D/DAP10 stimulation is shown to promote a sustained cancer progression via an increased energetic metabolism [55].

Cancer cells can drive immune suppression by multiple mechanisms, including the secretion of immune-suppressive cytokines and chemokines, such as TGF*β* and IL-10 [56], or FasL expressing microvesicles (TMV) which induce lymphocyte apoptosis [57]. The PI3K signaling is reported to mediate cellular responses upon exposure to these microenvironmental factors. The pleiotropic cytokine TGF*β*1 increases the expression of IL-10 and MCP-1 in melanoma cells, through a crosstalk between Smad, PI3K/AKT, and BRAF-MAPK signaling pathways. IL-10 induces decreased MICA expression on melanoma cells in an autocrine loop and blocks the antitumor functions of DCs and NK cells. MCP-1 recruits monocytes, which in turn secrete TGF*β*1, FGF, and proangiogenic factors (VEGF), and then differentiate into macrophages. The cooperation of these processes can boost the progression of melanoma [58, 59]. Cancer cells can also employ a more indirect mechanism to inhibit immune surveillance by enhancing the immune-suppressive function of T-regulatory (Treg) cells. TMV secreted by cancer cells can convert CD4(+)CD25(–) T cells into CD4(+)CD25(+)FOXP3(+) Treg, while increasing the expression by these cells of immune-suppressive factors, such as FasL, IL-10, TGF-*β*1, CTLA-4, granzyme B, and perforin [60]. *In vitro* studies demonstrate that the PI3K-mTOR pathway is required for the Granzyme B release by Treg, upon prolonged stimulation of TCR and CD28, synergically with IL-2 stimulation [61]. Moreover, Tregs derived from p110*δ* defective mice show an impaired suppression function *in vitro* and fail to secrete IL-10 [62].

A central role of PI3K in processes involving leukocytes motility (inflammation, adaptive immune responses, tumor infiltration) has been widely documented [63]. For example, PI3K isoform p110*γ* and p110*δ* are both required to mediate chemotaxis of NK cells induced by CXCL12 and CCL3 during pregnancy. In addition, p110*δ* is involved in S1P and CXCL10-mediated chemotaxis and in NK cell tissue distribution and tumor infiltration [3]. Antigen-activated p110*γ*-deficient CD4+ lymphocytes exhibit impaired F-actin polarization and migration into peripheral inflammatory sites in response to stimulation *ex vivo* with the CCR4 ligand CCL22 [64]. Using a mechanism PI3K dependent, cancer cells can also increase their malignancy by “emulating” some immune cell chemotactic responses. For example, the chemokine CCL5 (also called RANTES), previously known as a motility factor for some leukocytes during inflammation, can induce migration and metastasis of human cancer cells thanks to developing a *de novo* expression of CCL5 receptor (CCR5) at their surface, which is not present in non-cancerous cell lines. Tang et al. have demonstrated that chondrosarcoma cells express CCR5 and can sense CCL5 resulting in increased cell migration and metalloproteinases-3 secretion. The PI3K and NF-*κ*B pathways have been shown to play an essential role in this scenario [65].

## 4. Pharmacological Inhibition of PI3K in Cancer Treatment and Antitumor Immune-Response

The choice of suitable anticancer pharmacological agents requires a careful assessment of their side effects on the immune defense against cancerous cells. Although the role of a dysregulated PI3K pathway in the development of malignancy is well documented, a cancer treatment featuring PI3K inhibition might be deleterious to the immune response to tumors. In advanced renal cell cancer (RCC), treatment with Sorafenib but not Sunitinib can impair antitumor immune responses, through inhibiting PI3K and ERK phosphorylation in NK cells, thus, impeding the release by these cells of cytokines activating adaptive immune responses (i.e., IFN*γ*), as well as killing tumor cell targets [66]. However, this is in contrast with of antitumor immune enhancement effect reported for Sorafenib in hepatocellular carcinoma (HCC). This drug has been reported to downregulate the expression of metalloproteinase ADAM9 in HCC cells, which is involved in proteolytic cleavage of MICA, thereby, allowing this ligand to be displayed on the HCC cell surface for NK recognition [67]. A study by Ghebeh and coworkers provides evidence of detrimental effects arising from a combination of inhibition of the PI3K/AKT pathway and chemotherapy in an *in vivo* xenograft mouse model of cancer treatment. Indeed, the anthracycline doxorubicin has been shown to mediate nuclear translocation of the T-cell inhibitory molecule, B7-H1 (PD-L1, CD274), and phosphorylated AKT in breast cancer cells in a PI3K-dependent manner, restoring immune surveillance. Interestingly, these authors show an additional role for B7-H1 in preventing apoptosis in breast cancer cells, thus, providing a link between immune resistance and chemoresistance [68]. In CML therapy, in addition to diminishing the expression of ligands for the activating immunoreceptor NKG2D by tumor cells, the BCR/ABL-inhibitor Dasatinib can impair NK cell reactivity as well as IFN*γ* production. Dasatinib treatment was shown to inhibit the phosphorylation of PI3K and ERK, which are crucial for NK cell cytolytic activity [69]. The option of using p110 isoform-specific inhibitors for cancer treatment must be considered with care, as the function of a single isoform can be dually involved in promoting both tumor progression and antitumor immunity. A failure in NK cell-mediated clearance of cancerous cells has been reported in studies using p110*δ* knock-out mice. Although this isoform promotes the progression of leukemia, p110*δ* depletion results in a defective ability of NK cells to degranulate and kill a large variety of target cells [70]. Nevertheless, the use of p110*δ* inhibitor CAL-101 has recently proven effective in an *ex vivo* model of CLL, a disease that shows a high PI3K activity [71]. CAL-101 induces apoptosis of malignant cells without affecting normal T cells or NK cells. However, the effect of CAL-101 on NK or CD8+ and cell-mediated cytolytic functions of these cells has not yet been explored [72]. This evidence supports the notion that therapeutic benefits arising from targeting PI3K isoforms could depend on a balance between the benefit of purging cancer cells and the disadvantages of immunological impairment.

Evaluation of whether the inhibition of PI3K enzymes might lead to benefits in cancer therapy should also be based on the stage of disease when starting treatment. The sustained activation of lymphocytes in chronic inflammation, which underlies the development of several cancers, relies on PI3K activity in some cases. For example, p110*γ* isoform has been shown to drive the onset of colitis-associated tumors, due to its role in the activation and infiltration of myeloid cells and recruitment of T cells to the colon [73]. An anti-inflammatory therapy based on p110*γ* inhibition to prevent the onset of colitis-associated tumors could interfere with antitumor immunity when an early stage cancer is already developing, as the NK cells reactivity depends strongly on the activity of this isoform [3].

A quest for PI3K inhibitors with a selective action on malignant cells without affecting immune cells may reveal compounds that could offer a promising anticancer strategy while preserving anticancer immunological reactivity. For example, Honokiol, a plant-derived compound, was shown to be efficient in downregulating levels of phospho-S6 and B7-H1 in tumor cells via PI3K/mTOR pathway, thus, impairing the immune resistance of glioma, breast, and prostate cancer cell lines, while having no effect on critical proinflammatory T-cell functions. This does not occur with classic PI3K/mTOR inhibitors, including LY294002, wortmannin, AKT inhibitor III, and rapamycin [74]. Conversely, a selective therapy based on a specific pharmacologically induced T-cell PI3K/AKT pathway would prevent the tumor-induced death/suppression of immune cells potentially engaged in tumor clearance. Apoptosis induced *in vitro* on CD8(+) T-cells by tumor-derived microvesicles expressing FasL has been successfully inhibited by treating these lymphocytes with cytokine-based biologic agents, such as IRX-2, which, like IL-2, IL-7, or IL-15, block the apoptotic machinery through Akt activation [75].

## 5. Role of Immunomodulatory Drugs Currently Implemented for the Treatment of Tumor and Effect of PI3K Inhibitors on Immune Cells

A number of immunomodulatory drugs are currently under investigation for their anticancer activity. For instance, a novel strategy for treatment of advanced malignancies suggests the use of bispecific T-cell-engaging (BiTE) antibodies which cluster T-cells and cancer cells, and this results in an enhanced cytotoxic activity toward tumor cells. The recently developed therapeutic antibody Blinatumomab has a dual specificity for CD19 and CD3. Promising responses arose from the use of Blinatumomab in B-cell non-Hodgkin's lymphoma (NHL) and B-precursor acute lymphocytic leukemia (ALL) [76]. PF3512676 can activate TLR9 on plasmocytoid dendritic cells, thus, leading to increased expression of class I/II MHC costimulatory molecules and secretion of cytokines/chemokines that enhance antitumor NK cell activity. Lenalidomide can improve host immunity against tumor cells by stimulating LPS-induced IL-10 as well as costimulators of CD8+ T cells. Furthermore, it induces IL-2 and IFN*γ* delivery by T cells, resulting in activation of NK cells [77]. However, a hyperactive PI3K pathway in tumor cells can counteract the beneficial effects of immunomodulatory agents used for enhancing antitumor immune responses. p110*δ* isoform was shown to promote activation of CLL cells, as well as VEGF and FGF expression in response to lenalidomide [78]. With regard to VEGF and PI3 kinase downstream Signaling, it is worthy to mention that both VEGF and PI3 kinase inhibitors have an effect on the immune cells. Inhibitors and the main effects on the immune cells are summarized in Table 1.

Immunomodulators that enhance immune response against low immunogenic cancer-specific antigens during vaccine-mediated therapies are currently under development. One example is the use of multifunctional immunomodulator SA-4-1BBL during vaccination against the E7 HPV-associated oncoprotein for treatment of cervical cancer [87]. Another example is provided by IFN*α* that possess advantageous immunomodulatory properties including activation of DCs. However, the use of this chemokine in cancer immunotherapy is limited since it can cause autoimmune disorders [88]. Another strategy is to employ immune-directed (rather than antitumor) monoclonal antibodies (mAbs) targeting cytotoxic T-lymphocyte antigen-4 (CTLA-4), an inhibitory molecule on T cells. Ipilimumab and tremelimumab, two anti-CTLA-4 mAbs, have shown a better clinical antitumor response than the traditional tumor-targeting mAbs [89]. 

Immunomodulatory oligonucleotides (IMOs) represent a new class of compounds with anticancer properties. Their efficacy in inhibiting tumor formation has been demonstrated alone or in combination with chemotherapeutic agents both *in vitro* and *in vivo* in breast, prostate, and nonsmall cell lung cancer. TLR9 was recently found to be expressed in cancer cells apart from that in APCs. The anticancer activity of TLR9 as a receptor for IMOs and mediator of IMOs has also been described [90–92]. Thalidomide and its analogs inhibit angiogenesis indirectly by blocking the action of TNF-*α*, while activating costimulation in T cell. These drugs are employed alone or combined with chemotherapeutics in the treatment of some malignancies, including lung cancer and multiple myeloma [93, 94].

## 6. Concluding Remarks

Tumor growth may be the result of tumor proliferation and tumor-induced failure of immunity in killing cancer cells [95]. The PI3K signaling pathway is required in multiple processes, including not only cancer progression, escape of cancer cells from immunological surveillance, immune suppression and acquisition of leukocyte-like properties by cancer cells but also anticancer immune responses. This assumption raises concerns about the proper use of PI3K-targeting inhibitors. On one hand, the pharmacological inhibition of PI3Ks in cancer would be beneficial because of the blockage of tumor growth and immune-suppressive function mediated by PI3K. On the other hand, it could be hazardous since the PI3K signaling pathway is crucial in antitumor immunity. Therefore, to minimize deleterious effects, a therapeutic inhibition of PI3Ks should be selective as much as possible on targeting of cancer cells without having inhibitory effect on the immune system.

## Figures and Tables

**Table 1:** Main effects of the PI3K and VEGFR inhibitors on immune cells.

PI3K inhibitors	p110 isoform	VEGFR2/3 inhibitors	Effect	Ref.
PIK-75	*α*		Reduced production of TNF-*α* and IL-6, reduced expression of human endothelial cell adhesion molecules (E-selectin, ICAM-1, and VCAM-1), and human monocyte-endothelial cell adhesion.	[79]
AS-605240	*γ*		Reduced numbers of infiltrated proinflammatory macrophages and T cells.	[80, 81]
AS041164	*γ*		Reduced RANTES-induced chemotaxis/recruitment.	[82]
CAL-101	*δ*		Apoptosis of CLL cells and decreased production of various inflammatory and antiapoptotic cytokines by activated T cells.	[72]
IC87114	*δ*		Reduced antigen-induced airway infiltration of inflammatory cells, secretion of T(H)2 cytokines in lungs, and inhibition of monocytic integrin activation during diapedesis.	[83]
		SU5416	Reduced IFN*γ* secretion by CD4+ CD45RO+ T cells.	[84]
		E7080	Reduced lymphocytes in tumor.	[85]
		TSU68	Decreased expression of CXCL1 (by cancer cells) and IL-12 and reduced neutrophil migration into tumor.	[86]

## Abbreviations

PI3K: Phosphatidylinositol 3-kinase
NK: Natural killer
HLA: Human leukocyte antigen
KIR: Killer-cell immunoglobulin-like receptor
ILT: Immunoglobulin-like transcript
APC: Antigen presenting cell
IL: Interleukin
LPS: Lipopolysaccharide
TNF*α*: Tumor necrosis factor *α*
GM-CSF: Granulocyte macrophage colony-stimulating factor
DC: Dendritic cell
TLR: Toll-like receptor
mTOR: Mammalian target of rapamycin
MICA/B: MHC class I-related chain A/B
ULBP: UL16-binding protein
MCP-1: Monocyte chemoattractant protein-1
TGF*β*: Transforming growth factor *β*
CXCL: CXC chemokine ligand
CCL: CC chemokine ligand
CCR: CC chemokine receptor
ADAM9: Disintegrin and metalloproteinase domain-containing protein 9
IFN*γ*: Interferon *γ*
CML: Chronic myelogenous leukemia
CLL: Chronic lymphocytic leukemia
TMV: Tumor-derived microvesicles
VEGF: Vascular endothelial growth factor.

## References

[1] Cantley LC (2002). The phosphoinositide 3-kinase pathway. *Science*.

[2] Guillermet-Guibert J, Bjorklof K, Salpekar A (2008). The p110*β* isoform of phosphoinositide 3-kinase signals downstream of G protein-coupled receptors and is functionally redundant with p110*γ*. *Proceedings of the National Academy of Sciences of the United States of America*.

[3] Saudemont A, Garçon F, Yadi H (2009). p110*γ* and p110*δ* isoforms of phosphoinositide 3-kinase differentially regulate natural killer cell migration in health and disease. *Proceedings of the National Academy of Sciences of the United States of America*.

[4] Klippel A, Kavanaugh WM, Pot D, Williams LT (1997). A specific product of phosphatidylinositol 3-kinase directly activates the protein kinase Akt through its pleckstrin homology domain. *Molecular and Cellular Biology*.

[5] Currie RA, Walker KS, Gray A (1999). Role of phosphatidylinositol 3,4,5-trisphosphate in regulating the activity and localization of 3-phosphoinositide-dependent protein kinase-1. *Biochemical Journal*.

[6] Saito K, Scharenberg AM, Kinet JP (2001). Interaction between the Btk PH Domain and Phosphatidylinositol-3,4,5-trisphosphate Directly Regulates Btk. *Journal of Biological Chemistry*.

[7] Chang JS, Seok H, Kwon TK (2002). Interaction of elongation factor-1*α* and pleckstrin homology domain of phospholipase C-*γ*1 with activating its activity. *Journal of Biological Chemistry*.

[8] Milburn CC, Deak M, Kelly SM, Price NC, Alessi DR, Van Aalten DMF (2003). Binding of phosphatidylinositol 3,4,5-trisphosphate to the pleckstrin homology domain of protein kinase B induces a conformational change. *Biochemical Journal*.

[9] Sansal I, Sellers WR (2004). The biology and clinical relevance of the PTEN tumor suppressor pathway. *Journal of Clinical Oncology*.

[10] Kang S, Denley A, Vanhaesebroeck B, Vogt PK (2006). Oncogenic transformation induced by the p110*β*, -*γ*, and -*δ* isoforms of class I phosphoinositide 3-kinase. *Proceedings of the National Academy of Sciences of the United States of America*.

[11] Dbouk HA, Pang H, Fiser A, Backer JM (2010). A biochemical mechanism for the oncogenic potential of the p110*β* catalytic subunit of phosphoinositide 3-kinase. *Proceedings of the National Academy of Sciences of the United States of America*.

[12] Sun M, Hillmann P, Hofmann BT, Hart JR, Vogt PK (2010). Cancer-derived mutations in the regulatory subunit p85*α* of phosphoinositide 3-kinase function through the catalytic subunit p110*α*. *Proceedings of the National Academy of Sciences of the United States of America*.

[13] Shayesteh L, Lu Y, Kuo WL (1999). PlK3CA is implicated as an oncogene in ovarian cancer. *Nature Genetics*.

[14] Samuels Y, Wang Z, Bardelli A (2004). High Frequency of Mutations of the PIK3CA Gene in Human Cancers. *Science*.

[15] Samuels Y, Waldman T (2010). Oncogenic mutations of PIK3CA in human cancers. *Current Topics in Microbiology and Immunology*.

[16] Jia S, Liu Z, Zhang S (2008). Essential roles of PI(3)K-p110*β* in cell growth, metabolism and tumorigenesis. *Nature*.

[17] Lee SH, Poulogiannis G, Pyne S (2010). A constitutively activated form of the p110*β* isoform of PI3-kinase induces prostatic intraepithelial neoplasia in mice. *Proceedings of the National Academy of Sciences of the United States of America*.

[18] Dituri F, Mazzocca A, Lupo L PI3K class 1B controls the cell cycle checkpoint promoting cell proliferation in hepato-cellular carcinoma.

[19] Edling CE, Selvaggi F, Buus R (2010). Key role of phosphoinositide 3-kinase class IB in pancreatic cancer. *Clinical Cancer Research*.

[20] Guerreiro AS, Fattet S, Kulesza DW (2011). A sensitized RNA interference screen identifies a novel role for the PI3K p110*γ* isoform in medulloblastoma cell proliferation and chemoresistance. *Molecular Cancer Research*.

[21] Boller D, Schramm A, Doepfner KT (2008). Targeting the phosphoinositide 3-kinase isoform p110*δ* impairs growth and survival in neuroblastoma cells. *Clinical Cancer Research*.

[22] Lannutti BJ, Meadows SA, Herman SEM (2011). CAL-101, a p110*δ* selective phosphatidylinositol-3-kinase inhibitor for the treatment of B-cell malignancies, inhibits PI3K signaling and cellular viability. *Blood*.

[23] Sujobert P, Bardet V, Cornillet-Lefebvre P (2005). Essential role for the p110*δ* isoform in phosphoinositide 3-kinase activation and cell proliferation in acute myeloid leukemia. *Blood*.

[24] Cuní S, Pérez-Aciego P, Pérez-Chacón G (2004). A sustained activation of PI3K/NF-*κ*B pathway is critical for the survival of chronic lymphocytic leukemia B cells. *Leukemia*.

[25] Seliger B, Ritz U, Ferrone S (2006). Molecular mechanisms of HLA class I antigen abnormalities following viral infection and transformation. *International Journal of Cancer*.

[26] Groh V, Rhinehart R, Secrist H, Bauer S, Grabstein KH, Spies T (1999). Broad tumor-associated expression and recognition by tumor-derived *γδ* T cells of MICA and MICB. *Proceedings of the National Academy of Sciences of the United States of America*.

[27] Finn OJ (2008). Molecular origins of cancer: cancer immunology. *New England Journal of Medicine*.

[28] Borg C, Jalil A, Laderach D (2004). NK cell activation by dendritic cells (DCs) requires the formation of a synapse leading to IL-12 polarization in DCs. *Blood*.

[29] Trinchieri G (1995). Interleukin-12: a proinflammatory cytokine with immunoregulatory functions that bridge innate resistance and antigen-specific adaptive immunity. *Annual Review of Immunology*.

[30] Ikeda H, Old LJ, Schreiber RD (2002). The roles of IFN*γ* in protection against tumor development and cancer immunoediting. *Cytokine and Growth Factor Reviews*.

[31] Apte SH, Groves P, Olver S (2010). IFN-*γ* inhibits IL-4-induced type 2 cytokine expression by CD8 T cells in vivo and modulates the anti-tumor response. *Journal of Immunology*.

[32] Wendel M, Galani IE, Suri-Payer E, Cerwenka A (2008). Natural killer cell accumulation in tumors is dependent on IFN-*γ* and CXCR3 ligands. *Cancer Research*.

[33] Sasaki T, Irie-Sasaki J, Jones RG (2000). Function of PI3K*γ* in thymocyte development, T cell activation, and neutrophil migration. *Science*.

[34] Martin AL, Schwartz MD, Jameson SC, Shimizu Y (2008). Selective regulation of CD8 effector T cell migration by the p110*γ* isoform of phosphatidylinositol 3-kinase. *Journal of Immunology*.

[35] Ye BQ, Geng ZH, Ma L, Geng JG (2010). Slit2 regulates attractive eosinophil and repulsive neutrophil chemotaxis through differential srGAP1 expression during lung inflammation. *Journal of Immunology*.

[36] Fortin CF, Cloutier A, Ear T (2011). A class IA PI3K controls inflammatory cytokine production in human neutrophils. *European Journal of Immunology*.

[37] Haidinger M, Poglitsch M, Geyeregger R (2010). A versatile role of mammalian target of rapamycin in human dendritic cell function and differentiation. *Journal of Immunology*.

[38] Webb LMC, Vigorito E, Wymann MP, Hirsch E, Turner M (2005). Cutting edge: T cell development requires the combined activities of the p110*γ* and p110*δ* catalytic isoforms of phosphatidylinositol 3-kinase. *Journal of Immunology*.

[39] Kerr WG, Colucci F (2011). Inositol phospholipid signaling and the biology of Natural killer cells. *Journal of Innate Immunity*.

[40] Awasthi A, Samarakoon A, Dai X, Wen R, Wang D, Malarkannan S (2008). Deletion of PI3K-p85*α* gene impairs lineage commitment, terminal maturation, cytokine generation and cytotoxicity of NK cells. *Genes and Immunity*.

[41] Guo H, Samarakoon A, Vanhaesebroeck B, Malarkannan S (2008). The p110*δ* of PI3K plays a critical role in NK cell terminal maturation and cytokine/chemokine generation. *Journal of Experimental Medicine*.

[42] Wu J, Song Y, Bakker ABH (1999). An activating immunoreceptor complex formed by NKG2D and DAP10. *Science*.

[43] Upshaw JL, Leibson PJ (2006). NKG2D-mediated activation of cytotoxic lymphocytes: unique signaling pathways and distinct functional outcomes. *Seminars in Immunology*.

[44] Lanier LL (2008). Up on the tightrope: Natural killer cell activation and inhibition. *Nature Immunology*.

[45] Segovis CM, Schoon RA, Dick CJ, Nacusi LP, Leibson PJ, Billadeau DD (2009). PI3K links NKG2D signaling to a CrkL pathway involved in natural killer cell adhesion, polarity, and granule secretion. *Journal of Immunology*.

[46] Jiang K, Zhong B, Gilvary DL (2002). Syk regulation of phosphoinositide 3-kinase-dependent NK cell function. *Journal of Immunology*.

[47] Girart MV, Fuertes MB, Domaica CI, Rossi LE, Zwirner NW (2007). Engagement of TLR3, TLR7, and NKG2D regulate IFN-*γ* secretion but not NKG2D-mediated cytotoxicity by human NK cells stimulated with suboptimal doses of IL-12. *Journal of Immunology*.

[48] Ohtani M, Nagai S, Kondo S (2008). Mammalian target of rapamycin and glycogen synthase kinase 3 differentially regulate lipopolysaccharide-induced interleukin-12 production in dendritic cells. *Blood*.

[49] Utsugi M, Dobashi K, Ono A (2009). PI3K p110*β* positively regulates lipopolysaccharide-induced IL-12 production in human macrophages and dendritic cells and JNK1 plays a novel role. *Journal of Immunology*.

[50] Okkenhaug K, Wu L, Garza KM (2001). A point mutation in CD28 distinguishes proliferative signals from survival signals. *Nature Immunology*.

[51] Groh V, Wu J, Yee C, Spies T (2002). Tumour-derived soluble MIC ligands impair expression of NKG2D and T-cell activation. *Nature*.

[52] Boissel N, Rea D, Tieng V (2006). BCR/ABL oncogene directly controls MHC class I chain-related molecule A expression in chronic myelogenous leukemia. *Journal of Immunology*.

[53] Hemon P, Jean-Louis F, Ramgolam K (2011). MHC class II engagement by its ligand LAG-3 (CD223) contributes to melanoma resistance to apoptosis. *Journal of Immunology*.

[54] Noh KH, Kang TH, Kim JH (2009). Activation of Akt as a mechanism for tumor immune evasion. *Molecular Therapy*.

[55] Benitez AC, Dai Z, Mann HH (2011). Expression, signaling proficiency, and stimulatory function of the NKG2D lymphocyte receptor in human cancer cells. *Proceedings of the National Academy of Sciences of the United States of America*.

[56] Nicolini A, Carpi A (2009). Immune manipulation of advanced breast cancer: an interpretative model of the relationship between immune system and tumor cell biology. *Medicinal Research Reviews*.

[57] Whiteside TL (2006). Immune suppression in cancer: effects on immune cells, mechanisms and future therapeutic intervention. *Seminars in Cancer Biology*.

[58] Díaz-Valdés N, Basagoiti M, Dotor J (2011). Induction of monocyte chemoattractant protein-1 and interleukin-10 by TGF*β*1 in melanoma enhances tumor infiltration and immunosuppression. *Cancer Research*.

[59] Serrano AE, Menares-Castillo E, Garrido-Tapia M (2011). Interleukin 10 decreases MICA expression on melanoma cell surface. *Immunology and Cell Biology*.

[60] Szajnik M, Czystowska M, Szczepanski MJ, Mandapathil M, Whiteside TL (2010). Tumor-derived microvesicles induce, expand and up-regulate biological activities of human regulatory T cells (Treg). *PLoS One*.

[61] Efimova OV, Kelley TW (2009). Induction of granzyme B expression in T-cell receptor/CD28-stimulated human regulatory T cells is suppressed by inhibitors of the PI3K-mTOR pathway. *BMC Immunology*.

[62] Patton DT, Garden OA, Pearce WP (2006). Cutting edge: the phosphoinositide 3-kinase p110*δ* is critical for the function of CD4^+^CD25^+^Foxp3^+^ regulatory T cells. *Journal of Immunology*.

[63] Saudemont A, Colucci F (2009). PI3K signaling in lymphocyte migration. *Cell Cycle*.

[64] Thomas MS, Mitchell JS, Denucci CC, Martin AL, Shimizu Y (2008). The p110*γ* isoform of phosphatidylinositol 3-kinase regulates migration of effector CD4 T lymphocytes into peripheral inflammatory sites. *Journal of Leukocyte Biology*.

[65] Tang C-H, Yamamoto A, Lin Y-T, Fong Y-C, Tan T-W (2010). Involvement of matrix metalloproteinase-3 in CCL5/CCR5 pathway of chondrosarcomas metastasis. *Biochemical Pharmacology*.

[66] Krusch M, Salih J, Schlicke M (2009). The kinase inhibitors sunitinib and sorafenib differentially affect NK cell antitumor reactivity in vitro. *Journal of Immunology*.

[67] Kohga K, Takehara T, Tatsumi T (2010). Sorafenib inhibits the shedding of major histocompatibility complex class i-related chain a on hepatocellular carcinoma cells by down-regulating a disintegrin and metalloproteinase 9. *Hepatology*.

[68] Ghebeh H, Lehe C, Barhoush E (2010). Doxorubicin downregulates cell surface B7-H1 expression and upregulates its nuclear expression in breast cancer cells: role of B7-H1 as an anti-apoptotic molecule. *Breast Cancer Research*.

[69] Salih J, Hilpert J, Placke T (2010). The BCR/ABL-inhibitors Imatinib, nilotinib and dasatinib differentially affect NK cell reactivity. *International Journal of Cancer*.

[70] Zebedin E, Simma O, Schuster C (2008). Leukemic challenge unmasks a requirement for PI3K{delta} in NK cell-mediated tumor surveillance. *Blood*.

[71] Cuní S, Pérez-Aciego P, Pérez-Chacón G (2004). A sustained activation of PI3K/NF-*κ*B pathway is critical for the survival of chronic lymphocytic leukemia B cells. *Leukemia*.

[72] Herman SEM, Gordon AL, Wagner AJ (2010). Phosphatidylinositol 3-kinase-*δ* inhibitor CAL-101 shows promising preclinical activity in chronic lymphocytic leukemia by antagonizing intrinsic and extrinsic cellular survival signals. *Blood*.

[73] González-García A, Sánchez-Ruiz J, Flores JM, Carrera AC (2010). Phosphatidylinositol 3-kinase *γ* inhibition ameliorates inflammation and tumor growth in a model of colitis-associated cancer. *Gastroenterology*.

[74] Crane C, Panner A, Pieper RO, Arbiser J, Parsa AT (2009). Honokiol-mediated inhibition of PI3K/mTOR pathway: a potential strategy to overcome immunoresistance in glioma, breast, and prostate carcinoma without impacting T cell function. *Journal of Immunotherapy*.

[75] Czystowska M, Han J, Szczepanski MJ (2009). IRX-2, a novel immunotherapeutic, protects human T cells from tumor-induced cell death. *Cell Death and Differentiation*.

[76] Nagorsen D, Baeuerle PA (2011). Immunomodulatory therapy of cancer with T cell-engaging BiTE antibody blinatumomab. *Experimental Cell Research*.

[77] Corti A, Giovannini M, Belli C, Villa E (2010). Immunomodulatory agents with antivascular activity in the treatment of non-small cell lung cancer: focus on TLR9 agonists, IMiDs and NGR-TNF. *Journal of Oncology*.

[78] Herman SEM, Lapalombella R, Gordon AL (2011). The role of phosphatidylinositol 3-kinase-*δ* in the immunomodulatory effects of lenalidomide in chronic lymphocytic leukemia. *Blood*.

[79] Dagia NM, Agarwal G, Kamath DV (2010). A preferential p110*α*/*γ* PI3K inhibitor attenuates experimental inflammation by suppressing the production of proinflammatory mediators in a NF-*κ*B-dependent manner. *American Journal of Physiology*.

[80] Fougerat A, Gayral S, Gourdy P (2008). Genetic and pharmacological targeting of phosphoinositide 3-kinase-*γ* reduces atherosclerosis and favors plaque stability by modulating inflammatory processes. *Circulation*.

[81] Rodrigues DH, Vilela MDC, Barcelos LDS, Pinho V, Teixeira MM, Teixeira AL (2010). Absence of PI3K*γ* leads to increased leukocyte apoptosis and diminished severity of experimental autoimmune encephalomyelitis. *Journal of Neuroimmunology*.

[82] Ferrandi C, Ardissone V, Ferro P (2007). Phosphoinositide 3-kinase *γ* inhibition plays a crucial role in early steps of inflammation by blocking neutrophil recruitment. *Journal of Pharmacology and Experimental Therapeutics*.

[83] Lee KS, Park SJ, Kim SR (2006). Phosphoinositide 3-kinase-*δ* inhibitor reduces vascular permeability in a murine model of asthma. *Journal of Allergy and Clinical Immunology*.

[84] Basu A, Hoerning A, Datta D (2010). Cutting edge: vascular endothelial growth factor-mediated signaling in human CD45RO^+^ CD4^+^ T cells promotes Akt and ERK activation and costimulates IFN-*γ* production. *Journal of Immunology*.

[85] Ogino H, Hanibuchi M, Kakiuchi S (2011). E7080 suppresses hematogenous multiple organ metastases of lung cancer cells with nonmutated epidermal growth factor receptor. *Molecular Cancer Therapeutics*.

[86] Yamamoto M, Kikuchi H, Ohta M (2008). TSU68 prevents liver metastasis of colon cancer xenografts by modulating the premetastatic niche. *Cancer Research*.

[87] Sharma RK, Srivastava AK, Yolcu ES (2010). SA-4-1BBL as the immunomodulatory component of a HPV-16 E7 protein based vaccine shows robust therapeutic efficacy in a mouse cervical cancer model. *Vaccine*.

[88] Rizza P, Moretti F, Belardelli F (2010). Recent advances on the immunomodulatory effects of IFN-*α*: implications for cancer immunotherapy and autoimmunity. *Autoimmunity*.

[89] Fong L, Small EJ (2008). Anti-cytotoxic T-lymphocyte antigen-4 antibody: the first in an emerging class of immunomodulatory antibodies for cancer treatment. *Journal of Clinical Oncology*.

[90] Wang H, Rayburn ER, Wang W, Kandimalla ER, Agrawal S, Zhang R (2006). Immunomodulatory oligonucleotides as novel therapy for breast cancer: pharmacokinetics, in vitro and in vivo anticancer activity, and potentiation of antibody therapy. *Molecular Cancer Therapeutics*.

[91] Wang H, Rayburn ER, Wang W, Kandimalla ER, Agrawal S, Zhang R (2006). Chemotherapy and chemosensitization of non-small cell lung cancer with a novel immunomodulatory oligonucleotide targeting Toll-like receptor 9. *Molecular Cancer Therapeutics*.

[92] Rayburn ER, Wang W, Zhang Z, Li M, Zhang R, Wang H (2006). Experimental therapy of prostate cancer with an immunomodulatory oligonucleotide: effects on tumor growth, apoptosis, proliferation, and potentiation of chemotherapy. *Prostate*.

[93] Galustian C, Labarthe MC, Bartlett JB, Dalgleish AG (2004). Thalidomide-derived immunomodulatory drugs as therapeutic agents. *Expert Opinion on Biological Therapy*.

[94] Reck M, Gatzemeier U (2010). Targeted therapies: thalidomide in lung cancer therapywhat have we learned?. *Nature Reviews Clinical Oncology*.

[95] Koebel CM, Vermi W, Swann JB (2007). Adaptive immunity maintains occult cancer in an equilibrium state. *Nature*.

